# The Baffle Length Effects on the Natural Convection in Nanofluid-Filled Square Enclosure with Sinusoidal Temperature

**DOI:** 10.3390/molecules27144445

**Published:** 2022-07-12

**Authors:** Khaled Al-Farhany, Barik Al-Muhja, Farhan Ali, Umair Khan, Aurang Zaib, Zehba Raizah, Ahmed M. Galal

**Affiliations:** 1 Department of Mechanical Engineering, University of Al-Qadisiyah, Al Diwaniyah 58001, Iraq; khaled.alfarhany@qu.edu.iq (K.A.-F.); barik.almuhja@qu.edu.iq.com (B.A.-M.); 2 Department of Mathematical Sciences, Federal Urdu University of Arts, Sciences & Technology, Karachi 75300, Pakistan; farhanali.ali15@gmail.com (F.A.); aurangzaib@fuuast.edu.pk (A.Z.); 3 Department of Mathematical Sciences, Faculty of Science and Technology, Universiti Kebangsaan Malaysia (UKM), Bangi 43600, Malaysia; umairkhan@iba-suk.edu.pk; 4 Department of Mathematics and Social Sciences, Sukkur IBA University, Sukkur 65200, Pakistan; 5 Department of Mathematics, College of Science, King Khalid University, Abha 62529, Saudi Arabia; 6 Mechanical Engineering Department, College of Engineering, Prince Sattam Bin Abdulaziz University, Wadi Addawaser 11991, Saudi Arabia; ahm.mohamed@psau.edu.sa; 7 Production Engineering and Mechanical Design Department, Faculty of Engineering, Mansoura University, P.O. Box 35516, Mansoura 35516, Egypt

**Keywords:** baffle, nanofluid, natural convection, Rayleigh number, sinusoidal temperature distribution

## Abstract

The proper process of applying heat to many technological devices is a significant challenge. There are many nanofluids of different sizes used inside the system. The current study combines this potential to improve convection effects, considering numerical simulations of natural convection using Cu/water nanofluids in a square enclosure with bottom blocks embedded in baffles. The enclosure consists of two vertical walls with isothermal boundary conditions; the left wall is the sinusoidal heat source, whereas the right wall is cooled. The investigations dealt with the influences of nanoparticle concentration, Rayleigh number, baffle length, and thermal conductivity ratioon isotherms, stream functions, and average Nusselt number. The results present that, when the Rayleigh number rises, the fluid flow velocity increases, and the heat transfer improves. Furthermore, the baffle length case (L_b_ = 0.3) provides higher heat transfer characteristics than other baffle height cases.

## 1. Introduction

In many studies of heat transfer by natural convection, the low thermal conductivity of fluids such as air, oil, and water within the enclosure limits the thermal performance of the system and the heat transfer rate to the working fluid used in the system. It has very low thermal properties. A strong motivation to use fluids with a better thermal conductivity than conventional fluids has been developed to address this problem. Choi [1] demonstrated the use of unconventional ideas, such as nanoparticles floating in standard liquids, by adding small solid particles to the base liquid to create liquids with improved heat transfer properties, especially in thermal conductivity. 

Natural convection can be used for nuclear reactor cooling and thermal conditioning. It can be used to cool nuclear reactors, regulate the temperature of electronic equipment, and cool buildings and solar panels, in addition to various agricultural applications [2,3,4,5]. Natural convection has been extensively studied in air-filled enclosures with differently heated vertical walls, with different boundary conditions for the heat source walls and different boundary conditions of other walls. Researchers have studied a large number of square, rectangular, and inclined casings using nanofluids as working fluids, suitable for various wall conditions and no-thickness walls [6,7,8,9]. In addition, a large number of studies have been considered in recent years, such as [10,11,12,13,14,15,16,17,18,19], showing that introducing obstacles and wall-mounted baffles, filling the cavity with air, and varying the boundary conditions of the walls change the heat transfer. Mahmoudi et al. [20] considered the natural convection in square boxes with Cu/water nanofluids using statistical tests. The left wall exposed to a heat source baffle with continuous heat flow was mounted horizontally. According to the findings, the Nusselt number increased with *ϕ*. However, the results showed that it decreased with increasing baffle length (L_b_).

Furthermore, the temperature of the heating side declined with increasing Rayleigh number. Habibzadeh et al. [21] reported the natural convection of square boxes with Al_2_O_3_/water nanofluids. Insulating partitions were involved in the floor insulating boundaries of the cavity. In fact, the effects of many variables have been investigated, revealing that baffle height increases as the heat transfer rate decreases. Alternatively, Sayehvand et al. [22] examined natural convection in a square shell filled with Al_2_O_3_/water nanofluids using a numerical model. Two insulating baffles were mounted symmetrically on the horizontal walls of the housing. They concluded that increasing baffle length (L_b_) decreased the Nusselt number while causing the circulation core to move toward the vertical wall. Naoufalet al. [23] conducted further research to explore the effects of adding baffles to a nanofluid-filled square shell. At a vertical angle, the heating baffle is attached to the cavity’s bottom wall. The baffle length (L_b_) effects and its position (D_b_) were recognized. According to the findings, when the Rayleigh number increased, the average Nusselt number increased for every value of the volume fraction (*ϕ*). Furthermore, it was detected that the average Nusselt number reached its highest value as the baffle was put at the midpoint of the cavity.

Selimefendigil and Oztop [24] calculated the effect of different types of nanofluids (Al_2_O_3_/water and CuO/water) on natural convection for inclined square cavities. Baffles evenly divide the surface of the box with high material conductivity. The average Nusselt number increased with the Grashof number increases and decreased inversely with the position (D_b_). Due to its importance to many technical systems, the topic of natural convection in close fields with many types of boundary conditions and fixed wall thicknesses has recently received substantial attention from numerous scientists. Mobedi [25] statistically studied conjugated natural convection in inclined, square, gas-filled enclosures. The cavity contained two vertical conducting walls of finite thickness and uniform heat input, while the remaining walls remained thermally insulated. According to the findings, the horizontal walls had no direct effect on reducing the temperature difference between the enclosed walls. It decreased inside the cavity, especially when both thermal conductivity and Rayleigh number (Ra) were high. Zhou et al. [26] declared a statistical investigation of natural convective heat transport inside a rectangular cavity filled with a porous medium with exothermic properties. The enclosure consisted of two adiabatic horizontal walls; the left side was exposed to a heat source, while the right wall wascooled separately using a sinusoidal temperature distribution. The researchers concluded that the sinusoidal thermal condition and the phase deviation of the temperature profile had a positive influence on the natural convective heat transfer of the porous material in the enclosure. They also found that the increase in interphase heat transfer coefficient resulted in a faster drop in heat transfer.

Alsabery et al. [27] numerically studied natural convection inside a square enclose charged with Ag, Cu, Al_2_O_3_, or TiO_2_/water nanofluids exposed to sinusoidal temperature variation on the two horizontal walls and thermal insulation of the left and right vertical walls. They found that the heat transfer rate enhanced dramatically as the solid wall thickness increased. Al-Farhany and Abdulkadhim [28] investigated the conjugated natural convective heat transfer in a square porous cavity with partial heat on vertical and adiabatic horizontal walls. The authors concluded that increasing the Raleigh number and the thermal conductivity of the walls could control and facilitate heat transfer.

In another study (Zahanand Alim [29]), the conjugate heat transfer impact of a Cu/water nanofluid-filled thermally conductive vertical cavity was investigated numerically. The thick vertical wall on the left side of the box received stable heat flow, and the movable partition was connected with the bottom wall. The researchers found that the position of the baffles helped increase the rate of heat transfer. Nia et al. [30] numerically explored the amount of heat transfer of Cu/water nanofluids inside an L-shaped enclosure with baffles. The baffle location depended on the distance determined from the hot wall (AB). This study attempted to examine four alternative baffle configurations on the basis of baffle length (S) and distance from the left hot wall. Long baffles (L = 0.30 m) were proven effective in increasing the total Nusselt number. Furthermore, the blade with the S = 0.4 m position was more effective in increasing the amount of the Nusselt number compared to the S = 0.6 m case.

Keramat et al. [31] presented a numerical investigation of heat transport by natural convection in an H-shaped enclosure charged with Al_2_O_3_/water nanofluid. The top fin was attached to the hot sidewall, while the two sidewalls were maintained by cryostat, and the remaining walls were insulated. The influence of several variables such as Rayleigh number, baffle location within the cavity, and baffle boundary conditions on heat transfer in nanofluids was investigated. The conclusions presented that the position of the baffle had an effective influence on the amount of heat transfer, which was reduced when the baffle was placed on the top fin and increased when the baffle was placed on the bottom fin.

In tilted porous cavities with magnetic fields, Al-Farhany et al. [32,33] explored the fin positions and their effects on the natural convection of conjugated nanofluids. The data showed that increasing the Darcy number directly with the modified Rayleigh number and fin dimension increased the average Nusselt number. They also reviewed current research for natural convection in cavities of different shapes with internal hosts, such as the MHD effect and double diffusion [34,35,36,37,38,39,40,41].

According to the existing literature, no studies have focused on the effect of convectively connected baffles with finite size and sinusoidal temperature variation on the natural convection of many types of nanofluids. This problem can arise in a wide range of industrial applications, such as in collectors and electronic cooling devices that use nanofluids as working fluids.

The current paper presents a numerical study of the natural convective heat transfer and liquid flow of Cu/water nanofluids in a unit aspect ratio shell. High-conductivity single baffles were installed on the insulated horizontal walls of the cabinet of different lengths (L_b_). The hot wall on the left side of the enclosure had a sinusoidal temperature variation. In contrast, the cold wall on the opposite side had a constant wall temperature, and the remaining walls were adiabatic. This investigation deals with the influences of nanoparticle concentration, baffle length (L_b_), and Rayleigh number (Ra) on the rate of natural convection of nanofluids in a square cavity.

## 2. Numerical Technique

**Figure 1:**
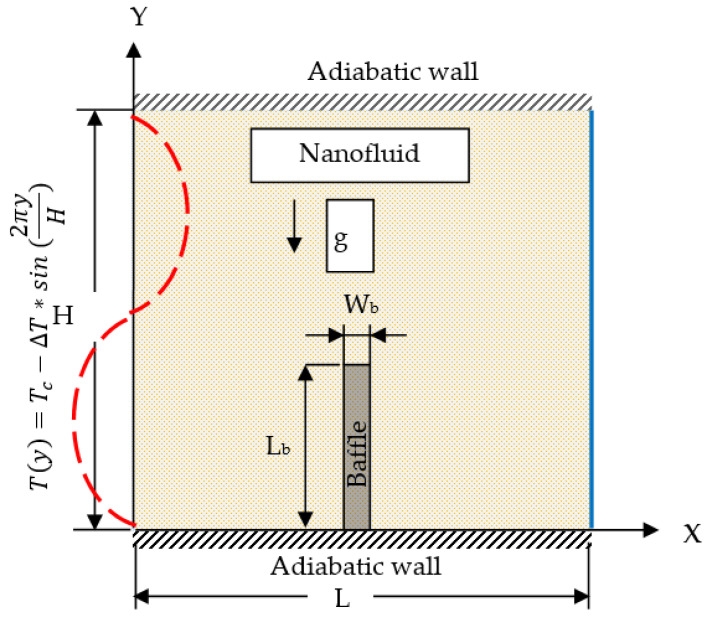
The geometry of the model.

A physical 2D enclosure with a square cross-section is shown in Figure 1. The left vertical wall has a sinusoidal temperature change when $\left(y\right)=T_{c}-\Delta T\times sin\left(\frac{2\pi y}{H}\right)$, whereas the opposite wall maintains a constant cold temperature (*T_c_*), and the other containment walls are adiabatic. The enclosed space is filled with a uniformly distributed Cu/water nanofluid. The baffle length (L_b_) and width (W_b_) of the highly conductive material baffle are dimensions fixed to the bottom wall. The temperature deviation is the controlling force for the flow of liquid in the enclosure. The Boussinesq approximation is used to define density changes.

The following dimensionless parameters can be used to write the governing equations for fluid mechanics and heat transport [42]:

(1) $$X=\frac{x}{L};\;Y=\frac{y}{H};\;U=\frac{uL}{\alpha_{f}};\;V=\frac{vL}{\alpha_{f}};\;P=\frac{pL^{2}}{\rho_{f}\alpha_{f}{}^{2}};\;\theta =\frac{T-T_{c}}{\Delta T};\;\;\alpha_{nf}=\frac{k_{nf}}{\rho_{nf}cp_{nf}};\;Pr=\frac{v_{f}}{\alpha_{f}};\;Ra=\frac{g\beta_{f}\left(\Delta T\right)L^{3}}{\alpha_{f}v_{f}}.$$

The cornerstones of the Boussinesq approximation and the dimensionless equations of fluid mechanics, such as the continuity, momentum, and energy equations for heat transfer through laminar natural convection under steady-state conditions are as shown below [43].

For fluid:

(2) $$\frac{\partial U}{\partial X}+\frac{\partial V}{\partial Y}=0.$$

(3) $$U\frac{\partial U}{\partial X}+V\frac{\partial U}{\partial Y}=-\frac{\partial P}{\partial X}+\frac{\mu_{nf}}{\rho_{nf}\alpha_{f}}\left(\frac{\partial^{2}U}{\partial X^{2}}+\frac{\partial^{2}U}{\partial Y^{2}}\right).$$

(4) $$U\frac{\partial V}{\partial X}+V\frac{\partial V}{\partial Y}=-\frac{\partial P}{\partial Y}+\frac{\mu_{nf}}{\rho_{nf}\alpha_{f}}\left(\frac{\partial^{2}V}{\partial X^{2}}+\frac{\partial^{2}V}{\partial Y^{2}}\right)+\frac{{\left(\rho \beta \right)}_{nf}}{\rho_{nf}\beta_{f}}RaPr\theta .$$

(5) $$U\frac{\partial \theta }{\partial X}+V\frac{\partial \theta }{\partial Y}=\frac{\alpha_{nf}}{\alpha_{f}}\left(\frac{\partial^{2}\theta }{\partial X^{2}}+\frac{\partial^{2}\theta }{\partial Y^{2}}\right).$$

For solid:

(6) $$\frac{\partial^{2}\theta_{w}}{\partial X^{2}}+\frac{\partial^{2}\theta_{w}}{\partial Y^{2}}=0.$$

The boundary conditions are applied in a dimensionless manner as described below.

All walls were assumed to be nonslip; therefore, the velocities used were *U* = *V* = 0. On the right sidewall, θ = 0. On the left sidewall, with sinusoidal temperature variations, $\theta =-\sin\left(2\pi Y\right)$. Both bottom and top walls were adiabatic $\left(\frac{\partial \theta }{\partial Y}=0\right)$. On baffle surfaces, $\frac{\partial \theta }{\partial X}\left|{}_{\mathrm{baffle}}\right.{=k}_{r}\frac{\partial \theta }{\partial X}\left|{}_{\mathrm{nf}}\right.$.

The thermophysical properties of the nanofluids were described in [44,45].

(7) $$\rho_{\mathrm{nf}}=\left(1-\phi \right)\rho_{f}+\phi \rho_{p}.$$

(8) $$\alpha_{\mathrm{nf}}=\frac{k_{\mathrm{nf}}}{{\left({\rho C}_{p}\right)}_{\mathrm{nf}}}.$$

(9) $${\left({\rho C}_{p}\right)}_{\mathrm{nf}}=\left(1-\phi \right){\left({\rho C}_{p}\right)}_{f}+\phi {\left({\rho C}_{p}\right)}_{p}.$$

The nanofluid effective dynamic viscosity, as defined by Brinkman, is expressed as follows [46]:

(10) $$\mu_{\mathrm{nf}}=\frac{\mu_{f}}{{\left(1-\phi \right)}^{2.5}}.$$

This formula may be used to calculate the nanofluid expansion coefficient.

(11) $${(\rho \beta )}_{\mathrm{nf}}=\left(1-\phi \right)\cdot {\left(\rho \beta \right)}_{f}+\phi \cdot {\left(\rho \beta \right)}_{p}.$$

The thermal conductivity of a nanofluid with spherical nanoparticles was calculated by Maxwell [47].

(12) $$k_{\mathrm{nf}}=k_{f}\frac{\left(k_{p}+2k_{f}\right)-2\phi \left(k_{f}-k_{p}\right)}{\left(k_{f}+2k_{p}\right)+\phi \left(k_{f}-k_{p}\right)},$$

where subscripts f and prefer to pure liquids and nanoparticles, respectively. Table 1 displays the thermophysical properties of the solid and water nanoparticles examined in this investigation [48].

The local Nusselt number $\left({\mathrm{Nu}}_{l}\right)$ at the hot boundary condition is defined by

(13) $${\mathrm{Nu}}_{l}={-\left.\frac{k_{\mathrm{nf}}}{k_{f}}\cdot \frac{\partial \theta }{\partial X}\right|}_{x=0}.$$

Integration of the local Nusselt number alongside the left sidewall can be used to predict the average Nusselt number $\left({\mathrm{Nu}}_{\mathrm{av}}\right)$.

(14) $${\mathrm{Nu}}_{\mathrm{av}}=\frac{1}{H}\int_{0}^{H}Nu_{l}.\;dY.$$

## 3. Solution Method

The finite element method (FEM) was used in the current study. It works by converting the formulation of the problem, such as the differential equations, into discrete problems (number of algebraic systems). In this study, the undetermined equations were approximated over the boundary. To obtain a convergent solution while minimizing truncation errors, the FEM employs methods distinct from calculus.

**Figure 2:**
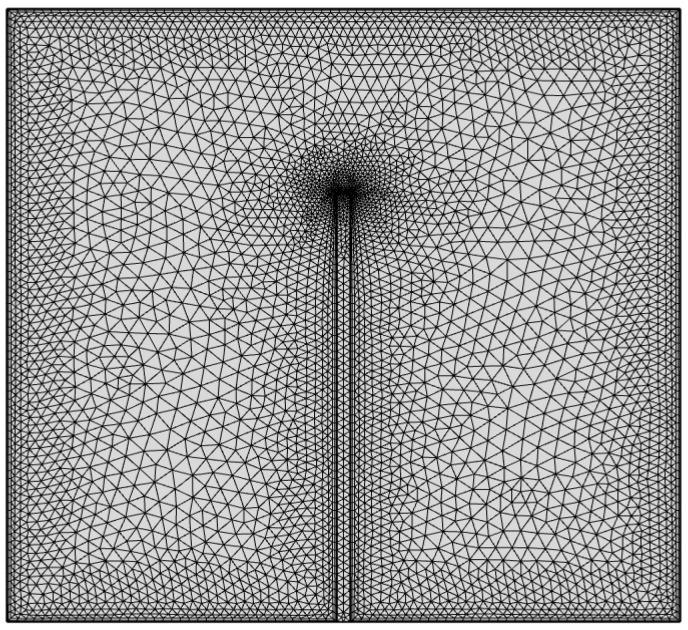
Triangle mesh distribution of the enclosure.

**Figure 3:**
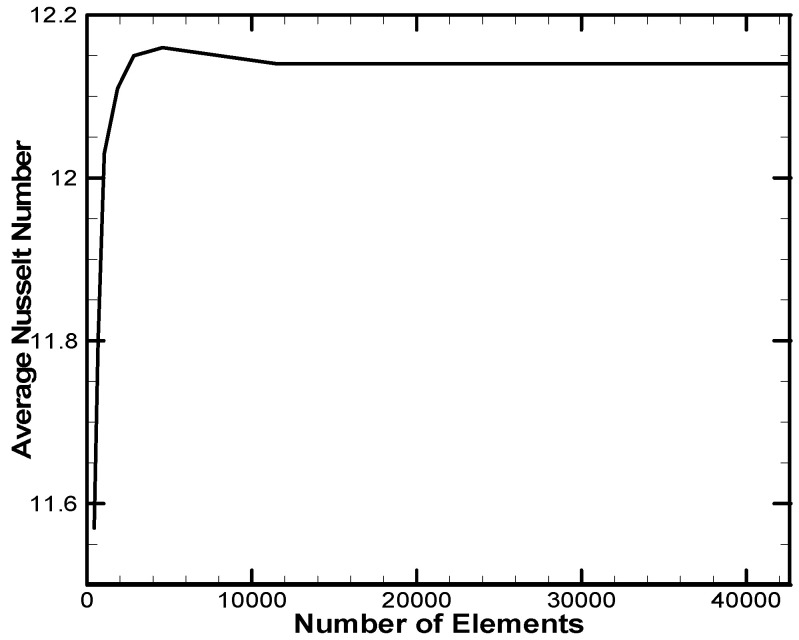
Convergence of the average Nusselt number along the left hot wall of the case.

Figure 2 displays that the 2D computational area for the baffle housing was divided into a multi element triangular mesh in a Cartesian system. Grid-independent studies were performed for different numbers of elements at Ra = 10^6^, L_b_ = 0.5, *ϕ* = 0.06. The accuracy of the results can be obtained by the precision of the average Nusselt number, which depends on the number of the mesh generation on the heated left sidewall of the shell using a nonuniform mesh. Figure 3 shows that several elements (approximately 11,500) gave accurate results without increasing the number of components.

**Figure 4:**
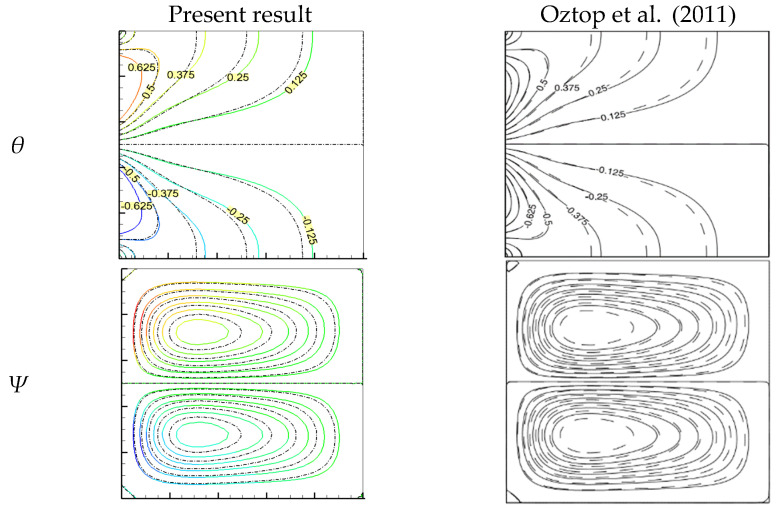
Comparison of isotherms $\left(\theta \right)$ and flow functions $\left(\psi \right)$ of Cu/water nanofluids at Ra = 10^4^, Kr = 1, and S=0 for *ϕ* = 0 water (solid line) and ϕ = 0.05 nanofluid (dashed line) [49].

**Figure 5:**
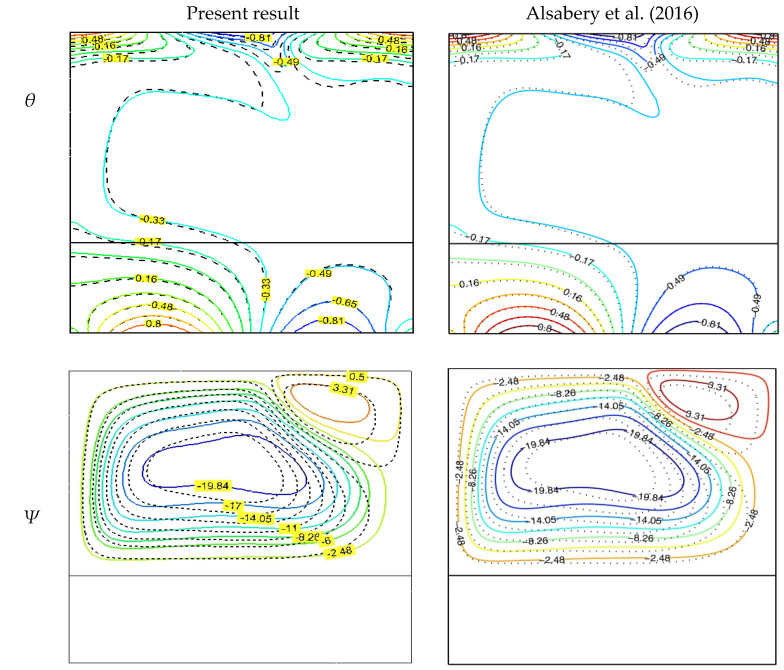
Comparison of isotherms $\left(\theta \right)$, flow functions $\left(\psi \right)$, and contours of Rayleigh number Ra = 10^6^ (*ϕ* = 0.1), thermal conductivity ratio Kr = 1, and wall thickness S=0.3 [27].

Finally, the proposed code was verified using the numerical results of Oztop et al. [49], which used the Rayleigh number (Ra = 10^5^) with a nanofluid concentration of about 0.05%. Oztop et al. updated these results in flow functions and isotherms. It was found to be quite accurate, as shown in Figure 4. The conjugated natural convection within the (Cu/water) nanofluid-filled shell and the limited thickness of the walls were also used to ensure the validity of the results. Figure 5 compares the current investigation’s findings to those of Alsabery et al. [27]. These results agree well with their previous work considering a Rayleigh number of 10^6^, nanofluid concentration of 0.1%, and effective wall thickness S of 0.3.

## 4. Results and Discussion

This computational investigation aimed to evaluate the natural convective heat transfer in a Cu nanoparticle/water-filled enclosure under sinusoidal (non-isothermal) boundary conditions, with vertical baffles attached to the lower horizontal wall. The enclosure had an adiabatic horizontal wall with a sinusoidal temperature variation on the left side wall and a constant cold temperature on the vertical right sidewall. The current study examined the effects of Rayleigh number (Ra), blade length (L_b_), blade thermal conductivity ratio (Kr), and solid volume fraction of nanoparticles (*ϕ*) on stream functions and isotherm patterns. Note that the Rayleigh number (Ra= 10^3^, 10^4^, 10^5^, and 10^6^), the solid volume percentage of nanoparticles (*ϕ* = 0, 0.02, 0.04, and 0.06), the ratio of thermal conductivity (K_b_ = 0.1, 1, and 10), and the baffle length (L_b_ = 0.3, 0.5, and 0.7) were varied for the current work, while the baffle thickness remained unchanged (W_b_ = 0.04).

### 4.1. Rayleigh Number Effects

**Figure 6:**
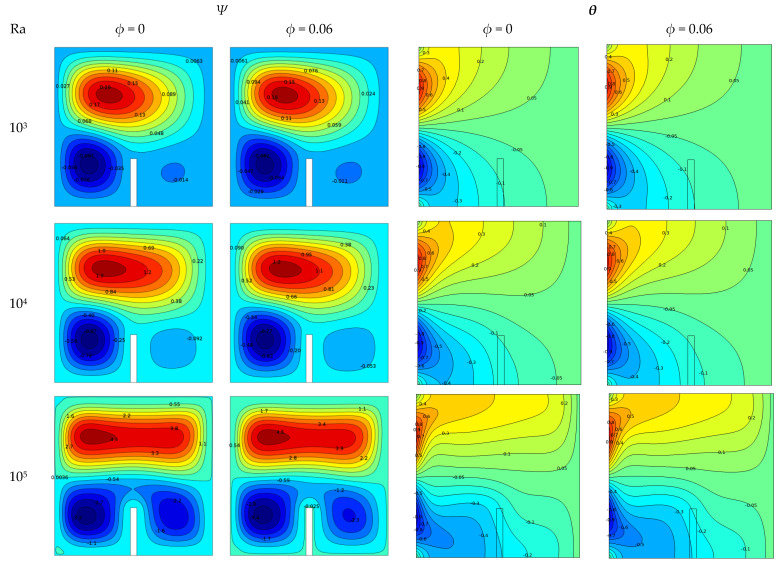

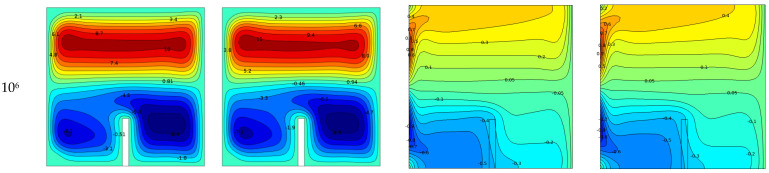
Effects of Rayleigh number on stream functions (left two columns) and isotherms (right two columns) at different nanoparticle solid volume fractions with Kr = 1 and L_b_ = 0.3.

Figure 6 illustrates the effects of Rayleigh number (Ra) and solid volume percentage of Cu/water nanofluid (0.06%) on stream functions and isotherms in the range 10^3^ to 10^6^, using K_b_ = 1 and L_b_ = 0.3 contours in a square cavity. Stream functions for pure and nanofluids are shown in the left two columns, while isotherms for pure and nanofluids are shown in the right two columns. Since the left wall of the enclosure exhibited non-isothermal temperature changes, it was heated by the upper half and cooled by the lower half in this part of the study.

Inside the case, three circular cores were created clockwise or counterclockwise. At low values (Ra = 10^3^), symmetrical flow and temperature distributions were found for all volume fraction values. The deviation of the isotherm was increasingly parallel to the right vertical wall, which is typical for conduction heat transfer. Despite Ra = 10^6^ increasing, the deviation became more parallel to the right vertical wall, the shell isotherm became a smooth curve parallel to the bottom wall, and the convective heat transfer became more controlling. When the Rayleigh number (Ra) increased, the strength of the buoyancy, as well as the strength of natural convection, increased. It can also be noted that the flow function value (flow strength) obtained increased the flow motion as the fluid was subjected to more heat. Both pure fluids and nanofluids reached maximum flow function values. At *ϕ* = 0, the stream function increased from 0.1 to 10, whereas, at *ϕ* = 0.6, the stream function increased from 0.16 to 11. Furthermore, the core strength of the stream function in the presence of nanofluids was greater than that in the presence of regular fluid (water). However, the presence of baffles bonded to the insulating wall under the casing restricted flow motion near the thermal wall, weakened the recirculation core, and affected the buoyancy.

**Figure 7:**
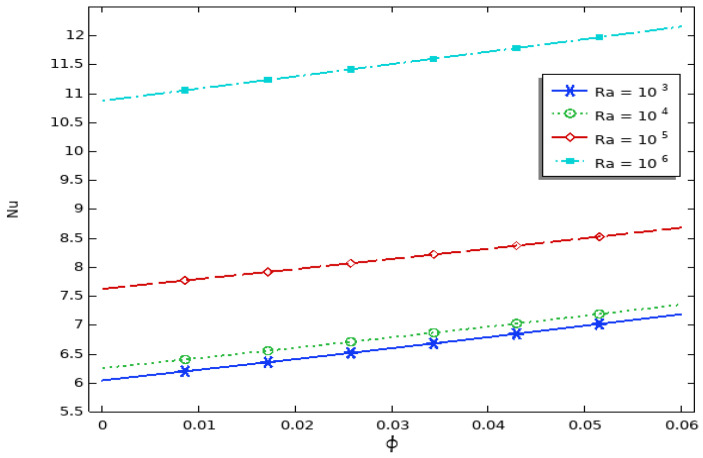
Average Nusselt numbers of different Rayleigh numbers for different solid volume fraction values with Kr = 1 and L_b_ = 0.3.

Figure 7 displays the Nu_av_ on the hot wall and the Rayleigh number for different concentrations of nanofluids. It can be noted that thermal conductivity increased directly with the volume fraction of the nanofluid. Furthermore, the average Nusselt number for different Rayleigh numbers was better than that for the pure base fluid. Moreover, the average Nusselt number was enhanced as the Rayleigh number increased due to larger flow function values and stronger buoyancy. Since the conduction was the essential mechanism of heat transfer, in this case, the effect of nanoparticles was visible at lower Rayleigh numbers. As the solid concentrations of nanofluids increased, the average Nusselt number and the heat transfer rate through the internal fluid increased. In addition, the convection effect of the Cu/water nanofluid was larger than that of water.

**Figure 8:**
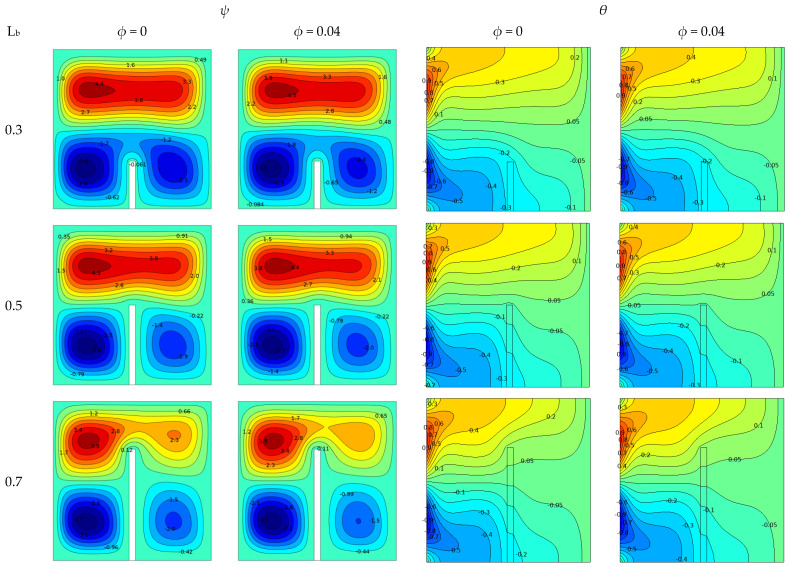
Effect of baffle length (L_b_) on different nanoparticle volume fraction values for stream functions (left two columns) and isotherms (right two columns) when Ra = 10^5^ and Kr = 10.

The baffle length and solid volume of the nanofluids at *ϕ* = 0.04, Kr = 10, and Ra = 10^5^ in the presence of vertical baffles for quadratic stream functions and isotherms are shown in Figure 8. The flow and thermal performance of the enclosure for three baffle lengths (0.3, 0.5, and 0.7) are shown. Stream functions for pure and nanofluids are shown in the left two columns, while isotherm profiles for pure and nanofluids are shown in the right two columns.

The two columns on the left illustrate the formation of asymmetric circulating cores flowing in three different directions within the casing. The primary was at the top, while the clockwise direction dominated. The length of the baffle had an important influence on the flow and shape of the flow cell. When a thin vertical baffle was placed in the middle of the insulating bottom wall, fluid flow was redirected and attenuated in the areas on either side of the baffle, affecting heat transfer at the wall. When the baffle length was L_b_ = 0.3, the flow function value at *ϕ* = 0.04 was *ψ* = 4.5, and the baffle had little effect on the flow. However, when the length of the baffle was L_b_ = 0.5, the value of the flow function was *ψ* = 4.4, and the baffle had little effect on the flow.

Furthermore, the value of the flow function decreased with increasing baffle length (L_b_ = 0.7). Because the blocking effect of a baffle is proportional to its height, a longer baffle yielded a greater blocking effect, and the value of the current function decreased to *ψ* = 4.4 when *ϕ* = 0.04. This increased the temperature in the area, resulting in a reduction in convective heat transfer.

The two columns on the right show isotherms to study the effect of baffles on the temperature distribution. The results revealed that, as the baffle length increased (L_b_ = 0.3–0.7), the flow restriction and resistance increased, resulting in a decrease in fluid flow and buoyancy on the upper left side of the casing. Thus, the temperature of the area increased, and the rate of heat transfer through the internal fluid decreased. The presence of baffles resulted in an asymmetric temperature distribution within the enclosure. In this case, the isotherms were practically uniform and parallel to the horizontal wall. It can be observed that the form of the isotherm close to the baffle also became further curved. As evidence, convection became the dominant mode of heat transport within the cage. In fact, the presence of nanoparticles affected the strength of liquid flow within the outer shell; as the concentration of the nanofluid increased, the thermal conductivity increased, the flow strength and buoyancy increased, and the rate of the heat transfer to the inner fluid increased.

**Figure 9:**
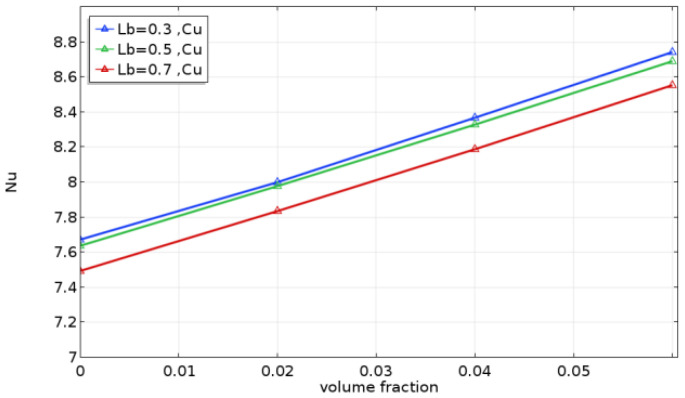
Average Nusselt number with volume fraction at different baffle lengths (L_b_) for Ra= 10^5^ and Kr = 10.

Figure 9 displays the influence of partition length (L_b_) on the average hot wall Nusselt number for various solid volume fractions. In general, the baffle length significantly affected the average Nusselt number. As the baffle length increased, the average Nusselt number decreased due to increased baffle blockage, resulting in a decrease in flow velocity and, therefore, a reduction in the heat transfer rate. Hence, it is clear that the average Nusselt number had a minimum at L_b_ = 0.7. It can also be shown that the average Nusselt number increased directly with the fluid flow velocity and heat transfer rates as the concentration of the nanofluids increased. Furthermore, the ideal heat transfer rate was similar to that of the nanofluid (*ϕ* = 0.06).

### 4.2. Baffle Thermal Conductivity RatioEffects

**Figure 10:**
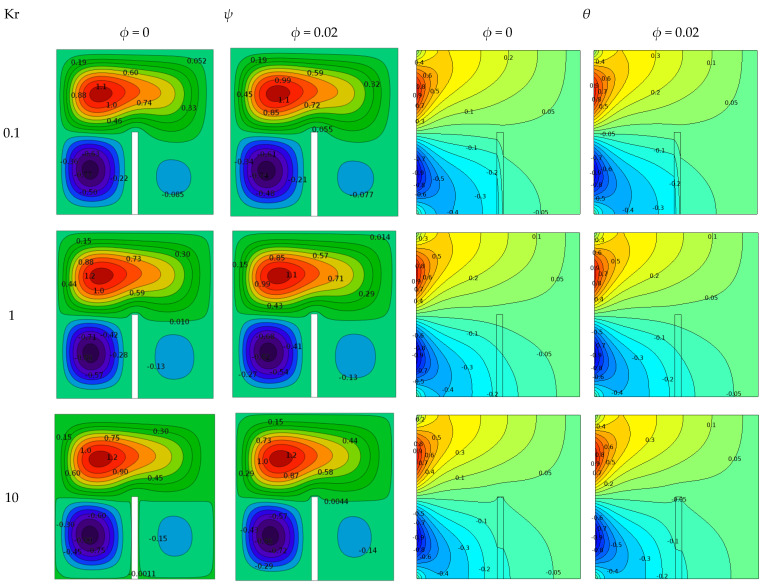
Effects of Kr on stream function (two left columns) and isotherms (two right columns) for different values of volume fraction at Ra = 10^4^ and baffle length = 0.5.

Figure 10 expresses the effect of varying the thermal conductivity of the stationary baffles on liquid flow and the thermal performance within the enclosure at various Cu/water nanofluid concentrations, with a sinusoidal (non-isothermal) temperature distribution at *ϕ* = 0.04, for Ra = 10^4^ and L_b_ = 0.5. Stream functions are shown in the left two columns, while the right two columns show the isothermal counters. Notably, an increase in the value of Kr indicated an increment in the thermal conductivity of the baffle material. When Kr = 0.1, low thermal conductivity led to high wall thermal resistance, resulting in a low heating intensity of the separator material and reduced heat transfer via conduction. This can be identified by the reduction in the isotherm gradient within the baffle thickness and the convection within the outer shell. The flow function value was 1.1 times that of the upper main recirculation core in the case of nanofluids.

Increasing the thermal conductivity ratio Kr from (0.1 to 10) resulted in a lower thermal wall resistance of the baffle while allowing the solid baffle to reflect more heat inside the cavity. In the case of nanofluids, the flow function value of the upper main recirculation core increased from *ψ* = 1.1 to 1.2 due to the increased heat flow through the baffle-fixed region, thereby increasing the elastic front. Furthermore, the distribution of isotherm counters near the solid–liquid interface and the slope of the temperature gradient changed significantly with thermal conductivity when the isotherm density was considered.

**Figure 11:**
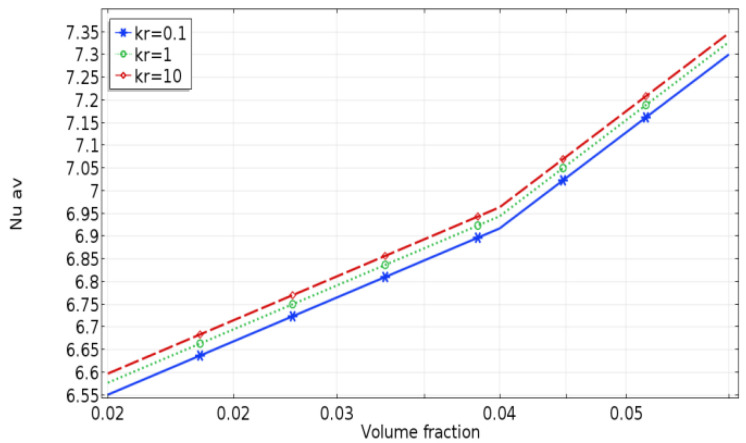
Average Nusselt number at different Kr, for different volume fractions with Ra = 10^4^ and L_b_ = 0.3.

Figure 11 displays the effect of different thermal conductivity ratios of the fixed baffles on the average Nusselt number for volume fractions with Ra = 10^4^ and L_b_ = 0.5. As shown above, increasing the solid volume fraction improved the heat transfer and convective motion. Additionally, as the thermal conductivity ratio (Kr) increased, the heat flux rate in the fluid domain also increased. Furthermore, it should be considered that the convective effect of the nanofluid was stronger than that of the base fluid at different baffle thermal conductivities and solid volume fractions.

### 4.3. Local Nusselt Number

**Figure 12:**
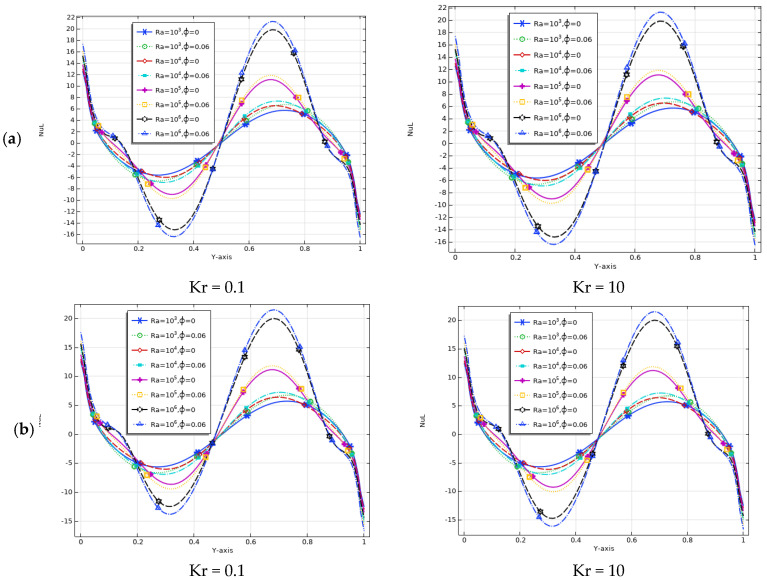

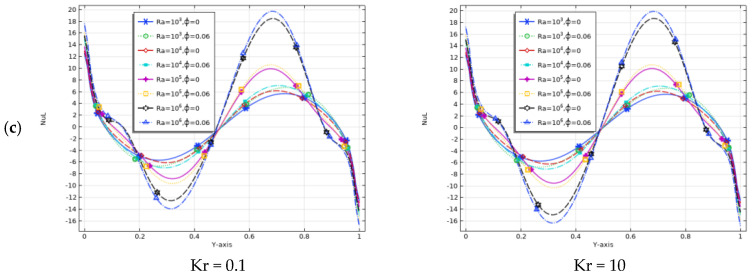
Local Nusselt numbers at the *Y*-interface for different Rayleigh numbers and nanofluid volume fractions with (**a**) L_b_ = 0.3, (**b**) L_b_ = 0.5, and (**c**) L_b_ = 0.7.

Figure 12a–c explain the fraction of local divergence of the Nusselt number $\left({\mathrm{Nu}}_{l}\right)$ at the interface location with the *Y*-axis indicating the Rayleigh number range, baffle length (L_b_), and volume (*ϕ*). Low heat transfer rates occurred because the internal fluid lowered the temperature of the entire enclosure space at a low Rayleigh number (Ra = 10^3^). A high Rayleigh number resulted in a significant increase in local convective heat transfer.

The local Nusselt number line above the coordinate (*Y*) had a sinusoidal shape, especially for large Rayleigh numbers (Ra = 10^4^ to 10^6^). As the Rayleigh number increased, this caused an increase in the local Nusselt number. This increase became significant around the vertical wall spacing (0.3 and 0.7) due to the sinusoidal variation of the boundary conditions. For pure water and nanofluids, the local heat transfer rate also increased significantly with decreasing baffle length. Additionally, as the volume percentage of nanoparticles increased, the heat transfer through the internal fluid within the cross-sectional area of the shell also increased. This was due to the increase in device thermal conductivity and targeted improvements in convective heat transfer. The localized Nusselt number increased along the left vertical interface wall, resulting from the presence of nanoparticles with the correct volume fraction. Cu/water nanofluids had higher local Nusselt numbers than water. In addition, an increase in thermal conductivity ratio further increased the heat flux in the solid–liquid section near the baffle. This increased the heat transfer, which significantly influenced the local Nusselt number.

In summary, at L_b_ = 0.3, Ra = 10^6^, and *ϕ* = 0.06, the maximum value appeared, while the minimum appeared at L_b_ = 0.7, Ra = 10^3^, and *ϕ* = 0. Therefore, it can be concluded that, when the baffle length (L_b_) was approximately 0.3, the heat transfer rate increased, whereas, in the range of 0.3–0.7, the heat transfer rate decreased.

## 5. Conclusions

This paper presented a numerical study of natural convection in a square enclosure filled with nanofluid subjected to sinusoidal temperature on the vertical walls with a vertical baffle fixed to the bottom wall. The study examined the effects of Rayleigh number (10^3^ ≤ Ra ≤ 10^6^), nanoparticle concentration (0.01 ≤ *ϕ* ≤ 0.06), baffle length (0.3 ≤ L_b_ ≤ 0.7), and thermal conductivity ratio (K_b_ = 0.1, 1, and 10) on the rate of natural convection of nanofluids in a square cavity. The following conclusions could be drawn:

1. When the baffle height increased, the natural flow direction was limited by the existence of the baffle. The isotherm distribution was better due to the larger convective heat transfer flux at lower altitudes.
2. When the baffle length was short (L_b_ = 0.3), the heat transfer rate increased, whereas, in the range of 0.5–0.7, the heat transfer rate decreased.
3. By increasing the thermal conductivity ratio, the Nusselt number increased due to the high heat transfer through the baffle, which could be transferred to the cavity without the baffle effect.
4. The Nusselt number increased with the Rayleigh number and nanofluid concentration due to the increase in buoyancy force and thermal conductivity. The circulating cores corresponding to nanofluids were also stronger than those of pure water at high Rayleigh numbers.

## Figures and Tables

**Table 1:** Thermophysical properties of fluid and nanoparticles at T = 25 °C [48].

Properties	Pure Water	Cu
**Cp (J/kg·K)**	4179	385
**ρ (kg/m^3^)**	997.1	8933
**k (W/m·K)**	0.613	401
**Β (1/K)**	21 × 10^−5^	1.67 × 10^−5^

## Data Availability

Not applicable.

## Nomenclature

AR	Aspect ratio
Cp	Specific heat, J·kg^−1^·K^−1^
d	Wall thickness, m
*g*	Gravitational acceleration, m·s^−2^
*k*	Thermal conductivity, W·m^−1^·K^−1^
*Kr*	Thermal conductivity ratio (*Kr* = k_b_/k_f_)
*L*	Length of the enclosure, m
*l_b_*	Baffle length, m
*L_b_*	Nondimensional Baffle length (*L_b_* = *l_b_*/H)
${\mathrm{Nu}}_{l}$	Local Nusselt number
Nu_av_	Average Nusselt number
*P*	Nondimensional pressure
*p*	Pressure, kg/(m·s^2^)
*Pr*	Prandtl number
*Ra*	Modified Rayleigh number
*Gr*	Grashof number
*T*	Dimensional temperature, °C
*U*	Nondimensional velocity component *X*-direction,
*V*	Nondimensional velocity component *Y*-direction,
*w_b_*	Baffle width, m
*W_b_*	Nondimensional baffle width, (*W_b_* = *w_b_*/L)
*X*	Nondimensional *X*-coordinate
*Y*	Nondimensional *Y*-coordinate
*Greek symbols*	
*ν*	Kinematic viscosity, m^2^/s
*ψ*	Stream function
*ϕ*	Solid volume fraction
*θ*	Dimensionless temperature
*Φ*	Baffle inclination angle, °
*ρ*	Density, kg/m^3^
*µ*	Dynamic viscosity, kg·m^−1^·s^−1^
*α*	Thermal diffusivity, m^2^·s^−1^
*β*	Thermal expansion coefficient, K^−1^
*Subscripts*	
b	Baffle
c	Cold
f	Fluid (pure water)
h	Hot
nf	Nanofluid
p	Nanoparticle
S	Solid
W	Wall
